# APP dyshomeostasis in the pathogenesis of Alzheimer’s disease: implications for current drug targets

**DOI:** 10.1186/s13195-024-01504-w

**Published:** 2024-06-29

**Authors:** Sònia Sirisi, Érika Sánchez-Aced, Olivia Belbin, Alberto Lleó

**Affiliations:** 1 Sant Pau Memory Unit, Neurology Department and Sant Pau Biomedical Research Institute, Hospital de la Santa Creu i Sant Pau, Universitat Autònoma de Barcelona, Sant Quintí 77, Barcelona, 08041 Spain; 2 Network Center for Biomedical Research in Neurodegenerative Diseases (CIBERNED), Madrid, Spain

**Keywords:** Alzheimer’s disease, Down syndrome, APP, Therapeutic targets, Drug

## Abstract

The Amyloid precursor protein (APP) is a transmembrane glycoprotein from which amyloid-β (Aβ) peptides are generated after proteolytic cleavage. Aβ peptides are the main constituent of amyloid plaques in Alzheimer’s Disease (AD). The physiological functions of APP in the human adult brain are very diverse including intracellular signaling, synaptic and neuronal plasticity, and cell adhesion, among others. There is growing evidence that APP becomes dysfunctional in AD and that this dyshomeostasis may impact several APP functions beyond Aβ generation. The vast majority of current anti-amyloid approaches in AD have focused on reducing the synthesis of Aβ or increasing the clearance of brain Aβ aggregates following a paradigm in which Aβ plays a solo in APP dyshomeostasis. A wider view places APP at the center stage in which Aβ is an important, but not the only, factor involved in APP dyshomeostasis. Under this paradigm, APP dysfunction is universal in AD, but with some differences across different subtypes. Little is known about how to approach APP dysfunction therapeutically beyond anti-Aβ strategies. In this review, we will describe the role of APP dyshomeostasis in AD beyond Aβ and the potential therapeutic strategies targeting APP.

## Introduction

Alzheimer’s disease (AD) is a neurodegenerative disorder characterized by accumulation in the brain of amyloid plaques and neurofibrillary tangles. The central hypothesis in AD revolves around the notion that amyloid plaques enhance the pathological aggregation of tau, which leads to increased neurofibrillary tangle formation, synaptic and neuronal loss. Under this paradigm, the amyloid-β (Aβ) peptide, the main constituent of amyloid plaques, has been conceptualized as one of the central therapeutic targets for disease modification in AD. The recent data from the clinical trials with lecanemab and donanemab in AD clearly support the rationale of anti-amyloid therapy and the important role of Aβ in disease pathogenesis. However, it is recognized that the pathophysiology of AD is much more complex and goes beyond the role of Aβ, or amyloid plaques. Amyloid pathology does not correlate well with cognitive deficits and there is a topographical mismatch between amyloid and tau pathology in the early stages of the disease. In addition, cell loss is not directly related to Aβ plaques or neurofibrillary tangles [1]. Although the topography of tau pathology is more closely associated with the clinical syndrome compared to amyloid pathology [2], synapse loss is the best neuropathological correlate of cognitive deficits in AD [3]. Brain resilience and the existence of other pathological comorbidities may explain some of these discrepancies, but it is also possible that the mismatch between AD pathology and clinical signs is due to other undetected/unnoticed changes related to AD pathophysiology.

One aspect that has not been fully addressed is the role of dyshomeostasis of the amyloid precursor protein (APP) in the pathogenesis of AD [4]. This conceptualization implies that the entire transmembrane protein is involved in AD pathogenesis, rather than the resultant proteolytic product (Aβ) only. APP dyshomeostasis can lead to an array of synergistic mechanisms beyond Aβ production and deposition that can independently contribute to neuronal and synaptic derangement. In this review we will elaborate on the basis for this paradigm and will discuss the implications for current and future therapeutic strategies. This review will not cover strategies aimed at targeting Aβ production or aggregation directly or indirectly that have been extensively reviewed [5].

## Genetics of APP in AD

Genetic data from autosomal dominant AD (ADAD) and Down Syndrome (DS) are the best examples of the causative role of APP in the disease pathogenesis. In patients with DS, an extra copy of the *APP* gene is believed to be the cause of the ultra-high risk of AD in this population [6]. In ADAD [7], more than 110 mutations have been described in *APP*, including missense mutations or duplications. Notably, a protective mutation (A673T) near the BACE1 cleavage site has been described [8]. The main mechanism by which these genetic alterations cause AD is believed to be a total or relative increase in the production of Aβ42 or an increase in the propensity to aggregate [8]. However, beyond the effects mediated by Aβ42, different studies have investigated the interference of these mutations with other APP functions that may also contribute to disease pathogenesis. Some ADAD-associated mutations are known to disturb α-secretase cleavage (K687N), interaction with Fe65 (Swedish mutation), and axonal transport (Swedish mutation, *APP* duplications). In particular, disruption of axonal transport seems to be a critical factor by which AD-mutations may induce disease. Intriguingly, the alterations in APP functions seem to depend on the specific amino acid substitution, as different substitutions on the same residue produce different effects [9].

Other genes closely related to *APP* have been consistently identified in genetic studies. Rare variants in *SORL1* have been found in families with early- and late-onset AD [10, 11]. SORL1 interacts with APP, and modulates its cellular trafficking through the secretase pathway. Variants in *SORL1* might alter APP trafficking along the secretory pathway interfering with the proteolytic APP pathway [12]. *ADAM10* is a major α-secretase that catalyzes APP ectodomain shedding in the brain [13]. Mutations in *ADAM10* (Q170H and R181G) have been identified in seven late-onset AD families [14] and attenuate α-secretase activity of ADAM10 and shift APP processing toward β-secretase-mediated cleavage, enhancing Aβ plaque formation and reactive gliosis [15]. These examples provide evidence that APP is closely involved in the pathogenesis of early and late-onset familial AD. However, the genetic architecture of sporadic AD is much more complex in which polygenicity and pleiotropy interact with multiple environmental factors. Genome-wide association studies of AD have identified at least 75 genomic loci that modify the risk of AD [16]. *APOE*ε4 is the major genetic risk factor identified so far, with multiple mechanisms associated with AD pathogenesis. However, most genetic variants individually have a small effect size, but in combination can contribute to a significant genetic risk. Interestingly, a genetic variant near the *APP* gene with impact on *APP* transcription has been reported [17]. Notably, recent exome sequencing data from more than 32,000 individuals implicates rare variants in genes related to APP pathways, such as ADAM10 or RIN3, providing evidence of a major role of APP processing in the pathophysiology of sporadic AD [18]. Taken together, these findings support a role of APP dysfunction not only in early and late-onset familial AD but also in common sporadic forms of AD. APP dysfunction in carriers of these variants is likely to involve several pathogenic mechanisms, many of which are unrelated to Aβ.

### APP dysfunction in AD

**Fig. 1:**
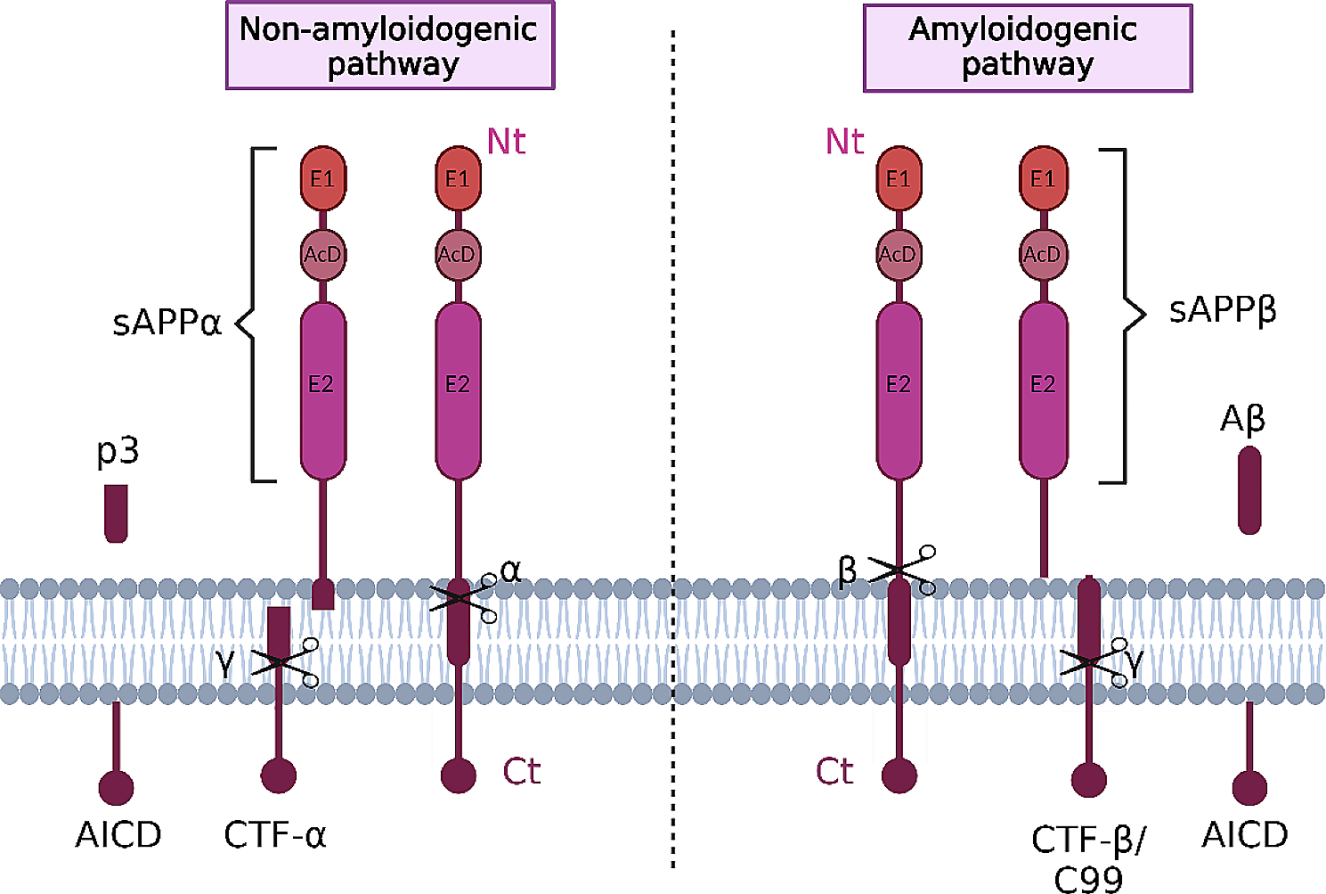
Canonical proteolytic pathways of APP. APP is formed by an extracellular N-terminal (Nt) domain [(divided into the E1 and E2 domains, linked by an acidic domain (AcD)], a transmembrane domain, and an intracellular C-terminal region (Ct). In the non-amyloidogenic pathway, the cleavage of APP is mediated by α- and γ-secretase, and the resultant products are sAPPα (soluble α-APP ectodomain) AICD (intracellular domain), and p3. In the amyloidogenic pathway, the cleavage of APP is mediated by β- and γ-secretase, and the resultant products are sAPPβ (soluble β-APP ectodomain), AICD and Aβ

Although the literature about the physiological functions of APP is overwhelming, its biological role is not fully understood. *APP* undergoes several alternative splicing events that generate APP mRNAs encoding isoforms from 365 to 770 amino acid residues. APP695 is the major isoform in the brain. Other encoding proteins include APP770, APP751, APP714 and APP639 [19, 20]. In the nervous system, APP has an important role in development early in the embryogenesis, mainly related to neuronal migration, synapse formation and plasticity, dendritic spine morphology and learning and memory [19, 20]. In the adult brain, APP modulates interactions with intracellular signaling pathways, and participates in the formation of axons and dendritic processes. APP is also deeply involved in the support of a variety of processes related to synaptic functions. APP binds to GABA_B_ receptors and regulates vesicular trafficking [21]. It is well established that during the intracellular transport, human APP can be processed via two proteolytic pathways: the amyloidogenic pathway, which leads to Aβ generation; and the non-amyloidogenic pathway, which leads to a fragment called p3 [22] (Fig. 1). APP can be cleaved by α- (non-amyloidogenic pathway) and β-secretases (amyloidogenic pathway) to generate two soluble fragments (sAPPα and sAPPβ, respectively), and subsequently by γ-secretase within the CTF region to generate p3, Aβ and APP intracellular domain (AICD) fragments [20] (Fig. 1). Therefore, it is likely that these processes become impaired in the AD brain, contributing to synaptic and neuronal derangement. Specific functions have been described for each of the APP proteolytic products generated in this pathway (see below).

### APP-derived metabolites other than Aβ

Different proteolytic products are generated from APP processing (Fig. 1) and these different fragments are implicated in different physiological or pathological functions.

**sAPP fragments**: sAPPα and sAPPβ are released to the extracellular space where they play a role in neuronal and synaptic processes. sAPPα is considered neuroprotective due to roles in synaptogenesis, neurite outgrowth and neuronal survival [23]. sAPPβ, in contrast, is considered less neuroprotective. sAPPβ is able to bind to GABA_B_ receptors and modulates synaptic transmission, and in excess, can be neurotoxic [24].

**p3**: this fragment is generated after α- and γ-secretase. p3 is non-neurotoxic, can be found in amyloid plaques, and is a major constituent of cerebellar preamyloid deposits in the brain of subjects with DS [24].

**C-terminal fragments (CTFs)**: after cleavage by α- and β-secretase, these transmembrane fragments interact with several adaptor and signaling proteins. Many of these interactions depend on the phosphorylation of APP residues [24]. Multiple phosphorylation sites in APP have been described in the C-terminal region of APP and some of these may be involved in the pathogenesis of AD and are more frequent in AD brains [25]. One of the most investigated phosphorylation is threonine (T) 668 [26]. This phosphorylation is common in AD and affects APP cleavage disturbing the cytoplasmic tail and the formation of CTFs [25]. These data support that post-translational modifications may be involved in the pathogenesis of the disease [25].

APP CTFs are implicated in interactions with motor proteins, such as kinesin, myosin and dynein to control axonal transport of vesicles. In fact, axonal swelling and transport defects are observed early in animal models of AD [27]. These data clearly suggest a physiological role of APP CTFs in signaling, and axonal transport.

There is compelling evidence in humans and animal models that the βCTF or APP-C99 is a contributor to AD pathogenesis [28]. The first evidence of accumulation of βCTF as a mechanism in AD was described in fibroblasts obtained from patients with DS [29]. Endosomal dysfunction in these cells depended on βCTF and not Aβ. βCTF accumulation has been confirmed also in human brain tissue. βCTF levels are increased in the brain in sporadic and familial AD cases [14, 30–32]. In addition, a study using Proximity Ligation Assay (PLA), a technique designed to detect molecules in close proximity, in AD brains showed that βCTF accumulation localized with tau-positive neurons in brain areas implicated in neurodegeneration [33]. Finally, a recent study indicates that βCTF accumulates in synapses in all forms of AD [34]. Several studies have investigated the role of βCTF accumulation in cellular or animal models. βCTF can be selectively neurotoxic to primary rat hippocampal neurons in culture [35] and capable of impair learning and working memory in vivo, in transgenic mice expressing βCTF, tg2576 APP mice and rats with CT105 peptide (a carboxyl-terminal fragment of APP) hippocampal microinjection [36, 37]. βCTF also accumulates early in neurons in specific AD-related brain areas in the 3xTg AD, the APP^E693Q^, the TgCRND8 and J20 mouse models [38–40] and in McGill-Thy1- APP rats [41]. In young C99-expressing mice, long-term potentiation is reduced and this reduction correlates with βCTF accumulation [42]. These electrophysiological abnormalities are rescued by β-secretase inhibition supporting a pathogenic role of βCTF in this model [38]. In an APP-transgenic mouse model with genetic inactivation of *PSEN1*, synaptic and cognitive deficits correlated with presynaptic APP-CTF accumulation [43]. It has also been described that intraneuronal accumulation of βCTF alters lysosomal and endosomal functions [42], activates microglia and astroglia [40, 42] and triggers mitochondrial structural, functional and mitophagy defects in AD models and in human brains [30, 31].

**AICDs**: this end-product from the two APP proteolytic pathways has been hypothesized to contribute to AD pathophysiology and mediate important signaling functions [44]. Y_682_ENPTY_687_ of AICD, a clathrin-mediated endocytosis motif, has been reported to interact with the Fe65 protein family (Fe65, Feb5L1), with Fe65 acting to stabilize AICD before translocation to the nucleus. Different neuroprotective and neurotoxic functions have been assigned to AICD, such as transcriptional activity, activation of GSK-3β to induce toxicity, trigger of degeneration in hippocampal neurons, induction of aberrant electrical activity and association with cognitive impairment [45].

Taken together, there is evidence that accumulation of βCTF and possibly other fragments in neurons and synapses could lead to multiple neurotoxic effects in lysosomal, endosomal, mitochondrial, and synaptic functions.

## Therapeutic implications

Anti-amyloid therapies aimed at clearing Aβ peptide have been a major focus of clinical trials for disease-modify therapies in the last two decades [46]. Drugs designed to inhibit β- or ɣ-secretase have been discontinued due to adverse effects linked to off-target effects on substrates other than APP. Currently, the most promising anti-amyloid strategy in AD is passive immunization. At least four second generation anti-Aβ antibodies have recently shown some signal of efficacy in patients with AD. Two of them, aducanumab and lecanemab, have been approved in the US for the treatment of AD and donanemab has shown efficacy in a phase 3 trial. There is some controversy about the efficacy, the magnitude of the effect, the length of treatment and the stage at which these drugs should be initiated. However, despite the controversy, these studies support the Aβ peptide as a relevant target for disease modification. It remains critical to determine whether reduction of large amounts of Aβ (~ 70% on average) is sufficient to obtain a clinically relevant effect over time. If Aβ overproduction or aggregation is one element of a broader APP dyshomeostasis, then it is possible that other therapeutic strategies aimed at restoring APP homeostasis and preventing accumulation of neurotoxic products other than Aβ may have a role in the therapeutic space. It is likely that each subtype of AD may induce a different degree or nature of APP dyshomeostasis depending on the main pathophysiological pathways. In this scenario there are different potential strategies (Table. 1) to restore APP homeostasis in AD depending on the underlying mechanism:

**Table 1:**
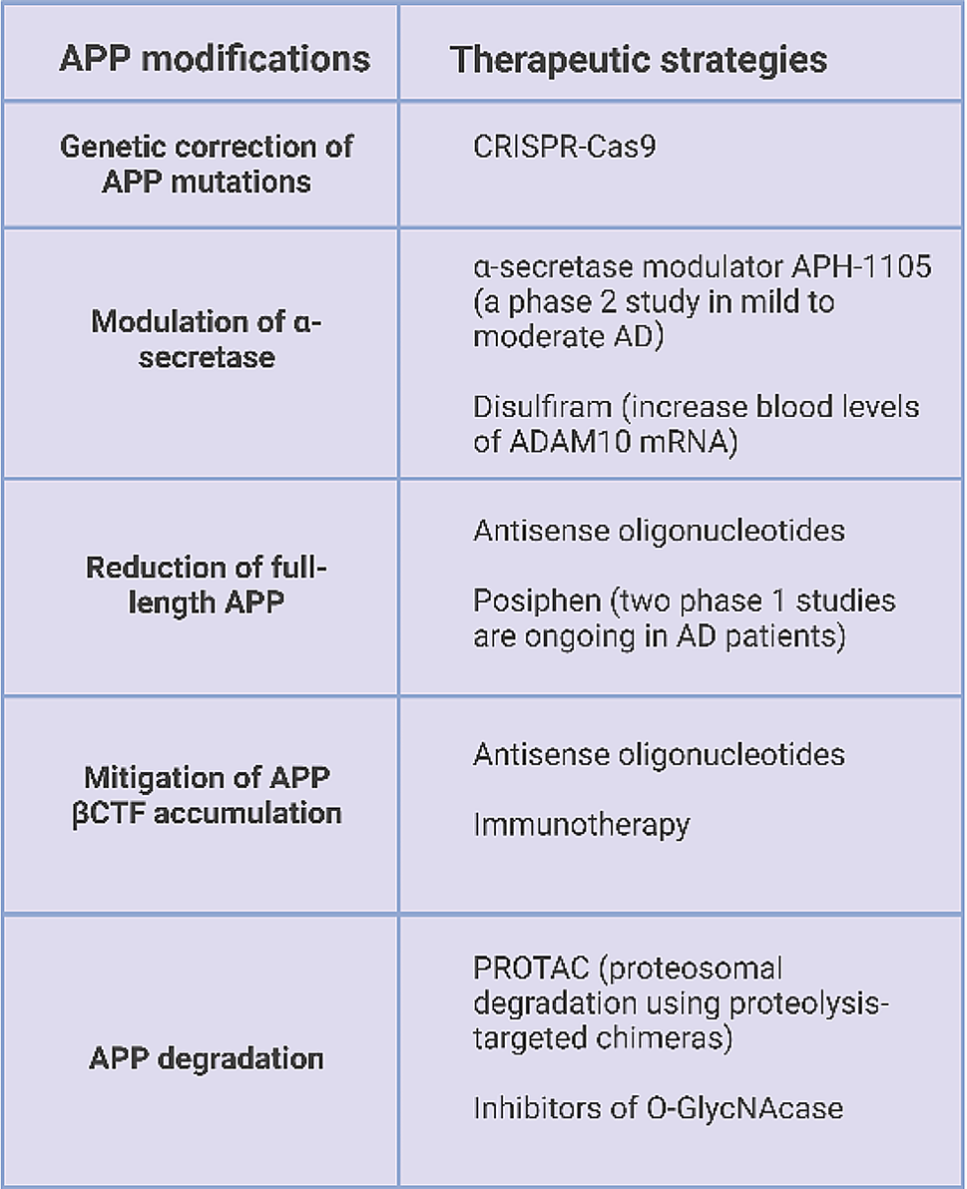
Summary of potential strategies to restore APP homeostasis in AD. APP modifications with the corresponding therapeutic strategies

- **Reduction of the expression of full-length APP**: downregulation of APP holoprotein could reduce all APP metabolites, including Aβ and βCTFs, and mitigate their effects on tau pathology and other downstream mechanisms. This approach is particularly attractive in DS or AD due to *APP* duplications, in which the main pathophysiological driver is increased gene dose. Posiphen, an oral small molecule that reduces translation of APP, has shown to normalize the levels of full-length APP and CTFs and to reduce Aβ species and phosphorylated tau in a mouse model of DS (Ts65Dn) [47]. There are no current trials with this molecule in DS. However, in a phase 1 clinical trial in healthy volunteers and patients with MCI (NCT01072812), treatment with posiphen reduced the levels of sAPP fragments and tau species in the CSF [48]. Two phase 1 studies in AD (NCT04524351 and NCT02925650) have been completed. In one study (NCT02925650) 10 patients received active treatment and 7 placebo (www.clinicaltrials.gov). The drug seemed to be well-tolerated and induced changes in CSF Aβ species. The other trial (NCT04524351) included 16 early AD patients, 10 treated with posiphen (80 mg) and 6 with placebo. No cognitive or biomarker data have yet been released. Another strategy to reduce APP expression consists of antisense oligonucleotide therapy. This approach can reduce synthesis of the entire APP holoprotein or exclude some exons by targeting APP mRNA. A study with an antisense oligonucleotide that reduces the synthesis of tau protein is currently in phase 1–2 in patients with AD (NCT03186989; NCT05469360). Experiments with APP antisense oligonucleotides have been shown to reduce APP expression in APP transgenic or SAMP8 mice and to improve learning and memory [49]. An antisense oligonucleotide that induces skipping of the APP exon required for proteolytic cleavage was also able to reduce Aβ in DS cell lines and transgenic mice [50]. A phase 1 trial using RNAi (RNA interference) for APP (ALN-APP, Alnylam Pharmaceuticals) in early onset AD has been completed (NCT05231785). The drug was generally well tolerated and induced sustained reductions in CSF concentrations of sAPPα and sAPPβ. In different human cell lines, RNAi with ALN-APP reduced APP βCTFs and restored endosomal defects (www.alnylam.com). The same compound is being investigated in patients with cerebral amyloid angiopathy. A trial with APP antisense oligonucleotides is planned in adults with DS. There are no registered studies using this approach in subjects with APP duplications. In these subtypes of AD this approach could restore the main pathway responsible of the disease. The effect of antisense oligonucleotides on the different APP splice variants is poorly understood.

- **Enhancement of APP degradation**: One potential approach to promote APP degradation is to enhance ubiquitination and proteasomal degradation using proteolysis-targeted chimeras (PROTAC) [51]. This system has been tested for tau and α-synuclein and could potentially be applied to APP metabolites. A potential application would be to target APP βCTFs, that are known to accumulate in neurons in patients with AD inducing defects at multiple subcellular levels. Another possibility is to treat with inhibitors of O-GlycNAcase (OGA), the glycoside hydrolase enzyme that removes O-linked N-acetylglucosamine (N-GlcNAc) from proteins. This strategy has shown to reduce the aggregation and toxicity of some proteins. OGA inhibitors have been initially investigated for tau-related diseases and two phase 1 trials in healthy volunteers have been completed (NCT04759365). A similar approach could be applied for APP.

- **Genetic editing of APP**: Some missense mutations in *APP* cause autosomal dominant AD. Mutations could be corrected using the CRISPR-Cas9 system early in life to prevent AD pathophysiology. CRISPR/Cas9 correction in neurons with a *PSEN2* N141I mutation normalized the Aβ42/40 increase and abolished the electrophysiological deficits. A similar approach could be applied to *APP* mutations [52].

- **Modulation of α-secretase**: sAPPα has neuroprotective actions and promotes synaptogenesis. A potential strategy in AD is to increase the proteolysis of this fragment. In addition to increased synaptogenesis, activation of α-secretase may reduce amyloidogenic processing of APP. A phase 2 trial with the retinoid acitrecin, an ADAM10 activator, showed an increase in CSF sAPPα levels in 22 patients with AD [53]. The compound disulfiram, which is used for alcohol dependence, can also activate ADAM10 and it has been shown to increase blood levels of ADAM10 mRNA [54]. There are no studies with disulfiram in AD patients. A phase 2 study in mild-to-moderate AD with the α-secretase modulator APH-1105 is currently ongoing (NCT03806478).

- **Mitigation of APP βCTF accumulation**: APP βCTF accumulates in the brain in all forms of AD [31, 33, 34], and this fragment contributes to neuronal and synaptic derangement [28]. Consequently, a potential strategy would be to specifically reduce APP βCTF. This could be achieved through immunotherapy. There are some antibodies that target specifically βCTF [55] that have been tested in animal models. Currently, there are no trials following this approach.

## Future perspectives

In light of the increase in the investment for AD research, it is critical to further consider other targets beyond Aβ and tau. One target that has gained little attention is APP itself. Instead of the current dogma where Aβ plays a solo role in APP dyshomeostasis, a wider view can place APP at the center stage. In this scenario, the field would benefit from novel therapeutic approaches aimed at restoring APP dysfunction in concert with anti-Aβ therapies. The most direct application of an APP-restorative approach would be to downregulate the synthesis of APP through antisense oligonucleotide therapy in individuals with *APP* duplications or with DS. In these AD subtypes this strategy could restore the main causative driver of AD pathology. The application of this strategy to cases without *APP* gene dosage changes remains more speculative as the effects of APP downregulation on physiological APP functions are uncertain. Subjects with missense *APP* mutations could be treated using gene therapy with CRISPR-Cas9 to correct the mutation early in life using adenovirus. The approach in sporadic AD poses a more complex scenario because much less is known about the contribution of APP dyshomeostasis to the disease. However, risk variants in the *APP* gene have been reported in sporadic AD in some GWAS studies [17] and APP accumulates in AD in neurons prone to neurodegeneration [33, 34] suggesting a contributing role in sporadic AD as well. In this common form of AD, a combination therapy using different strategies to restore APP homeostasis together with immunotherapy against Aβ could be envisioned if these approaches show benefit individually. It is also likely that some specific subgroups of sporadic AD may show enhanced APP dyshomeostasis, and may be eligible for intervention. This scenario is in line with a personalized medicine approach, in which different subgroups of sporadic AD are treated with different strategies directed at specific pathophysiological pathways.

## Conclusions

Despite the evidence that APP becomes dysfunctional in AD, APP dyshomeostasis has not been fully approached from a therapeutic perspective beyond anti-Aβ interventions. Under this wider paradigm more studies are needed to investigate the degree and nature of APP dysfunction in different subtypes of AD, and how to approach therapeutically APP dysfunction beyond anti-Aβ. This holistic view could yield new strategies to enrich the therapeutic arsenal for this devastating disease.

## Data Availability

Not applicable.

## Abbreviations

APP: Amyloid Precursor Protein
AD: Alzheimer’s Disease
ADAD: Autosomal Dominant Alzheimer’s Disease
AICD APP: APP Intracellular Domain
Aβ: Amyloid-β peptide
CTFs: C-terminal fragments
DS: Down Syndrome
N-GlcNAc: O-linked N-acetylglucosamine
OGA: O-GlycNAcase
PLA: Proximity Ligation Assay
PROTAC: Proteolysis-Targeted Chimeras
sAPPα: Soluble APPα ectodomain
sAPPβ: Soluble APPβ ectodomain
